# High-output bending motion of a soft inflatable microactuator with an actuation conversion mechanism

**DOI:** 10.1038/s41598-020-68458-5

**Published:** 2020-07-21

**Authors:** Satoshi Konishi, Hirotoshi Kosawa

**Affiliations:** 1 Department of Mechanical Engineering, College of Science and Engineering, Ritsumeikan University, Kusatsu, 525-8577 Japan; 2 Graduate Course of Science and Engineering, Ritsumeikan University, Kusatsu, 525-8577 Japan; 3 Ritsumeikan Global Innovation Research Organization, Ritsumeikan University, Kusatsu, 525-8577 Japan

**Keywords:** Nanoscience and technology, Mechanical engineering

## Abstract

The improvement of soft inflatable microactuators using an actuation conversion mechanism is presented in terms of high-output generation; a bending inflatable microactuator with the conversion mechanism is designed to generate high-output bending motion. The designed microactuator consists of a pneumatic balloon on a base film and a conversion film over the balloon and ribs on the backside of the base film. A conversion film converts the inflating motion of a pneumatic balloon into a bending motion. The fabricated microactuator with a pneumatic balloon of 13 mm in diameter is 16 mm × 40 mm × 850 μm. A 25 μm thick polyimide film is used as a conversion film over the pneumatic balloon because polyimide film is both non-stretchable and flexible. An array of Si ribs (15 mm × 40 mm × 400 μm) is integrated on the backside of the base film. Analysis of the microactuators with and without the conversion mechanism indicates that the output performance is improved with the addition of the conversion mechanism, as designed. As a result, the microactuator with the conversion film generates a maximum force of 1.72 N at 80 kPa, whereas the microactuator without the conversion film generates a maximum force of 0.15 N at 40 kPa. The improved microactuator can provide 4.2 mN/mm^3^ as the force density. In addition to fundamental characterization, the performance characteristics of the actuators are examined by combining the fundamental results.

## Introduction

The miniaturization of tools for in vivo diagnosis and surgical operation has achieved remarkable development. Compact and safe tools improve minimally invasive medicine in areas of a body with a limited leeway. Various forceps are used to treat organs or to perform a biopsy in a body. Endoscopes have been continuously improved and miniaturized to enhance the quality of life of patients. Microelectromechanical systems (MEMS) or micromachine technology as well as traditional precise machining are anticipated to provide smaller devices by means of high precision and high throughput micromachining based on semiconductor production technology. MEMS is based on thin film technology developed in the manufacturing process of semiconductor devices. The advantage of a thin structure for a medical tool is that it can be inserted through a small incision or attached on the surface of existing medical tools. Flexible MEMS devices, such as implantable electrodes^1–4^ and drug delivery devices^5–7^, have been studied in the MEMS field. Reported implantable flexible electrodes were composed of thin film metal (gold/chromium) on polyimide film^1–3^. The neurostimulator^4^ used a parylene-metal skin technology. A capacitive pressure sensor for intraocular pressure monitoring in a mouse eye used parylene and liquid crystal polymer^5^. Micropumps for drug delivery were developed using polydimethylsiloxane (PDMS)^6^ and platinum/titanium on parylene^7^. Wearable sensors^8–12^ are another attractive application of flexible MEMS. For instance, a flexible and wearable biosensor used a polypropylene membrane^8^ and a skin-mountable strain sensor used nanotubes-Ecoflex nanocomposites^9^. Flexible MEMS devices have introduced polymers in addition to Si which is the principal material traditionally used in MEMS. Furthermore, many studies on stretchable electronics have been reported for conventional electronics as well as flexible MEMS^13^. A cuff actuator^2^ was reported to hold and keep a condition of flexible multi-electrodes for stimulating and recording of nerve signal. It is attractive possibility for MEMS technology to be able to provide both sensors and actuators in the same process. In parallel to flexible sensor development, various soft microactuators have been reported with polymers^14,15^. The review paper^14^ classified elastic actuators into membrane, balloon, bellows and artificial muscle actuators. Pneumatic balloon actuator fabricated by MEMS technology based on the lithography^16^ and bending balloon microactuator using electro-conjugated fluids^17^ were classified in the balloon type.

The review paper^14^ compared the force density of microactuators including balloon actuators. The pneumatic balloon actuator^16^ (16 mm × 16 mm × 800 μm) could generate 0.05 N at 20 kPa. The bending balloon microactuator^17^ (4.5 mm in diameter and 20 mm long) using electro-conjugated fluids generated 0.9 mN. They generated lower force density than hydraulic actuator and electrostatic actuator but higher than piezoelectric microactuator according to the review^14^. In comparison, so-called pouch motor (75 mm × 25 mm × 100 μm) was recently reported to generate 0.2 N^18^, which showed the higher force density than other traditional microactuators. The review paper^15^ categorized elastic inflatable actuators^16,18–29^ by manufacturing techniques: Full lithography^24^, 2D molding^20,27^, 3D molding^18,19,21,23,25,26,28,29^, and additive manufacturing^21,22^. The manufacturing techniques is closely related with the performance of elastic inflatable actuators. Soft lithography as well as full lithography were used for 2D molding^20,24,27^. The review paper^15^ employed the bending coefficient instead of the force density because most works reported deformations than forces. The bending motion of different elastic inflatable actuators were compared using a bending coefficient which is the ratio of curvature over input pressure. It was found that a thin film actuator fabricated by two-dimensional molding had a higher bending coefficient than other types.

**Figure 1:**
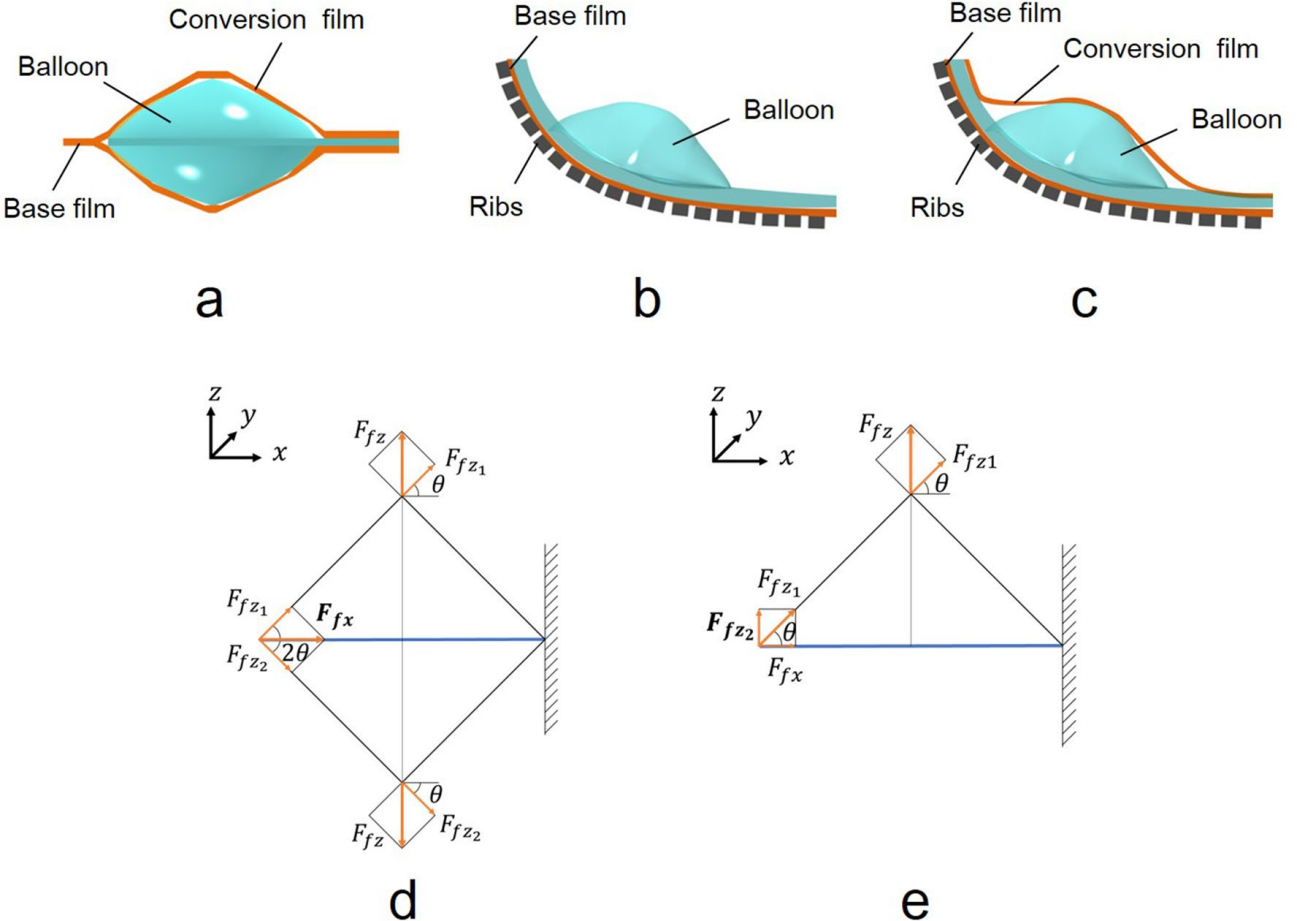
Different types of bending-type PBAs as thin film soft inflatable microactuator. (**a**) Inflatable microactuator with a conversion mechanism for the contracting motion^33^. (**b**) First generation of bending-type PBA^16^, (**c**) Inflatable microactuator with a conversion mechanism for the bending motion^34^. (**d**) Force vector graph of the conversion mechanism for the contracting motion microactuator illustrated in (**a**). (**e**) Force vector graph of the conversion mechanism for the bending motion microactuator depicted in (**c**). The force F_fz_ represents the pushing force against the conversion film caused by the inflated balloon.

The authors have continuously developed various types of pneumatic balloon actuators (PBAs) using MEMS-based thin film technology over the last 20 years^16,30–33^. Bending-type PBAs, twisting-type PBAs, and contracting-type PBAs were developed. The cuff actuator for functional electrical stimulation can be regarded as expansion-type PBA. Bending-type PBAs were first developed using MEMS technology. As mentioned above^15^, thin film bending-type PBA is a highly efficient elastic inflatable actuator. The first generation of bending-type PBAs bends toward the balloon. All PDMS PBAs, which are the third generation of bending-type PBAs, contain a cavity between two bonded films that exhibit different mechanical properties^30^. A PDMS film with a low stiffness deforms more easily than a film with a high stiffness. Consequently, the PBA structure bends to the side of the high stiffness film. Twisting-type PBAs are composed of layered structures of bending-type PBAs^31^. Contracting-type PBAs can generate a linear motion by converting the inflating motion of the balloon into a contracting motion. We have developed two types of contracting-type PBA. One is a telescopic paired PBA^32^. A paired bending-type PBA converts inflatable motion into bending motion and then telescopic motion. Two bending-type PBAs bonded at their edges bend by pressurization in the opposite direction. A paired PBA converts the opposite bending motion of two PBAs into telescopic motion, linear contracting-motion. The other employs a pantograph conversion mechanism for contracting-type PBA in order to directly convert inflating motion into linear contracting motion as illustrated in Fig. 1a^33^. This contracting-type PBA can generate linear expansion and contraction by a pantograph structure holding balloons, which is composed of two polyimide films. The film for the pantograph mechanism requires both non-stretchability and flexibility. Previously, the linear motion possible via contracting-type PBAs was applied to drive wire-driven forceps and a bending endoscope^33^. The contracting-type PBA using a pantograph mechanism was developed to make the best use of inflatable motion of pneumatic balloon. It is effective to improve the expansion characteristics of a pneumatic balloon itself for the actuator. Materials for the balloon membrane were examined from this point of view. A different kind of silicone rubber, KE-1606 (Shin-Etsu Chemical Co., Ltd.) was selected because KE-1606 was superior to PDMS in terms of elongation performance. As results of comparison between KE-1606 and PDMS, a single contraction-motion PBA using KE-1606 showed attractive force generation. The maximum generated force by contracting-type actuator using KE-1606 achieved 2.5 N (110 kPa) while an actuator using PDMS generated 1.2 N (80 kPa).

This paper addresses a conversion mechanism for the high-output bending motion of soft inflatable microactuators. Bending-type PBA reported in 2000^16^ showed 0.24 mN / mm^3^ as the force density whereas the pouch motor^29^ reported higher performance than that of the bending-type PBA^16^ after fifteen years. This paper reports the improvement design of bending-type PBA by the conversion mechanism. Figure 1 compares the motion principles of different types of PBAs, including an inflatable microactuator with a conversion mechanism for the contracting motion (Fig. 1a)^33^, a first-generation bending-type PBA (Fig. 1b)^16^, and an inflatable microactuator with a conversion mechanism for the bending motion (Fig. 1c)^34^. The force F_fz_ in Fig. 1 represents the pushing force against the conversion film caused by the inflated balloon. This paper focuses on the conversion mechanism for the inflatable microactuator in Fig. 1c with the aim of providing high-output bending motion. Force vector graphs are shown with side views of the microactuators in Fig. 1. Figure 1a shows the actuation conversion mechanism for the contracting-type PBA. A pair of pneumatic balloons is arranged at the top and bottom of a base film of an actuator. Conversion films placed over the pneumatic balloons in the top and bottom of the base film can convert the inflating motion of the balloons to a contracting motion. The conversion films form a pantograph-like shape during contraction. Contracting-type PBAs can generate high force by efficiently transmitting the inflating motion of the balloons. It was reported that a single actuator could generate 1.2 N at 80 kPa from 6 mm × 6 mm × 400 µm device (with a 5 mm × 5 mm balloon). Figure 1b shows a first-generation bending-type PBA. This PBA has a PDMS balloon on a polyimide base film integrated with Si ribs on the backside of a base film. The ribs can prevent unnecessary deformation except that in the longitudinal direction. Bending-type PBAs were designed to be applied to an organ exclusion tool for endoscopic surgery, during which at least sub-Newton-force is required to lift and exclude organs. The conversion films are designed to transmit pneumatic actuation effectively. This paper presents a conversion mechanism for the high-output bending motion of soft inflatable microactuators. Figure 1c illustrates the proposed conversion mechanism for the bending motion of the microactuator^34^. A conversion film is added to cover over the pneumatic balloon in the composition of the first-generation bending-type PBA in Fig. 1b. In contrast with the conversion mechanism for the contracting microactuator with a pair of balloons, the inflating motion of a single balloon on a base film is converted to the bending motion. Figure 1d, e depict force vector graphs in details of the conversion mechanism. The pantograph mechanism in Fig. 1d converts the inflating motion into the contraction motion where force is converted from F_fz_ to F_fx_. The conversion mechanism for the bending motion in Fig. 1e can be regarded as an upper half structure of the pantograph structure, where ribs are additionally designed. F_fz1_ is converted to F_fz2_ by the actuation conversion mechanism in Fig. 1e so that F_fz2_ generates bending motion. This paper examines the design, fabrication, and characterization of proposed conversion mechanism for the high-output bending motion of soft inflatable microactuators by extending the first report^34^ on a preliminary study of the conversion mechanism.

## Results

### Fabrication results of the bending microactuator with the conversion mechanism of actuation

**Figure 2:**
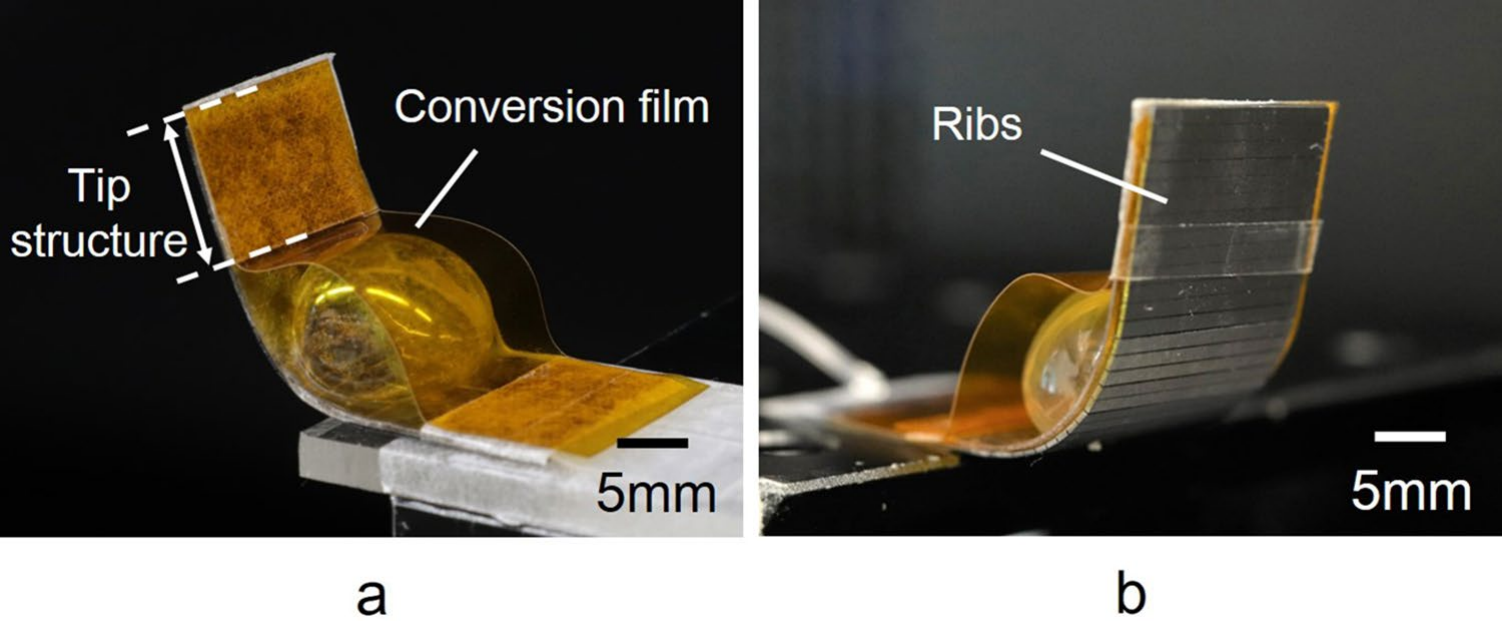
Fabrication results of bending motion microactuator with conversion mechanism. (**a**) Photograph of the frontside of the microactuator. (**b**) Photograph of backside of the microactuator. Fabricated micoractuator was 16 mm × 40 mm × 850 μm. The tip of the microactuator was 10 mm from a bonding part of the films in the photograph. The microactuator bends up when a circular pneumatic balloon (13 mm in diameter) is inflated by pressurization. A 25 μm thick polyimide film was used as a conversion film over the pneumatic balloon. An array of Si ribs (15 mm × 40 mm × 400 μm) were integrated under a base film, where ribs (950 μm wide) were arrayed with 50 μm of intervals.

**Figure 3:**
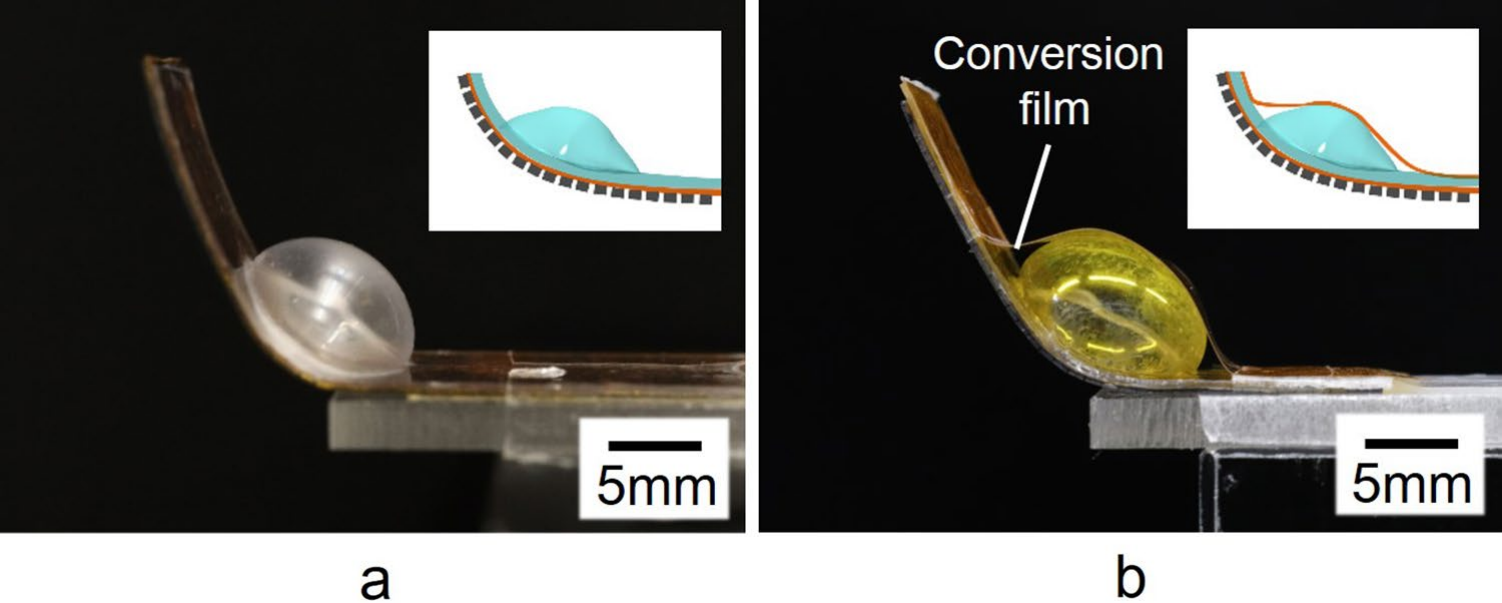
Side views of two different types of bending microactuators. (**a**) First generation of bending-type PBA ^16^. (**b**) Inflatable microactuator with a conversion mechanism for the bending motion^21^. (**a**, **b**) correspond to Fig. 1b,c as shown in illustrations in the top right corner of each photographs. Both bending microactuator have ribs under the base film. The bending microactuator in (**b**) has the actuation conversion film in addition.

A bending inflatable microactuator was designed with the conversion mechanism of actuation to generate a high-output bending motion. The conversion mechanism converts the inflating motion of a pneumatic balloon to a bending motion using a conversion film. Figure 2 shows the fabrication results of the bending microactuator (16 mm × 40 mm × 850 μm) with the conversion mechanism using a conversion film and ribs. Figure 2a, b show photographs of the front and back of the microactuator. The bending microactuator bends up when a pneumatic balloon (13 mm in diameter) is inflated by pressurization. The pneumatic balloon was designed to have a circular shape, not a rectangular shape, in plain view to generate vertical deformation effectively. A polyimide film (25 μm thick) was used as a conversion film over the pneumatic balloon. The conversion film should be both non-stretchable and flexible. Si ribs (15 mm × 40 mm × 400 μm) were integrated under a base film (25 μm thick polyimide film). Si is suitable for ribs because it is a rigid material that can be micromachined. Si ribs prevent unnecessary deformation, except longitudinal bending motion, without losing the flexibility required for the bending motion. A base film with ribs is flexible in one direction, toward the top side of the film. A base film is stiff in the other direction in which the ribs are arranged because ribs spaced by narrow gaps are contacted at their edges and locked. Locked ribs inhibit the deformation of a base film. Si ribs reinforce the stiffness of a base film to withstand heavy weight. The gap between ribs was 50 μm in this study, and each rib was 950 μm wide. Figure 2 shows that a polyimide conversion film can convert the inflating motion of the pneumatic balloon into the bending motion of the microactuator (see Supplementary video). Figure 3 shows photographs of two different types of bending microactuators corresponding to Fig. 1b, c. This paper evaluates the proposed actuation conversion mechanism for the bending motion in Fig. 1c through the comparison between the two types. Figure 3a shows the first-generation bending-type PBA with a pneumatic balloon on a base film, which corresponds to Fig. 1b. The Si ribs were fabricated under a base film. Figure 3b shows the microactuator integrated with a conversion film for bending motion and the ribs under the base film, which corresponds to Fig. 1c.

### Characterization of actuator

**Figure 4:**
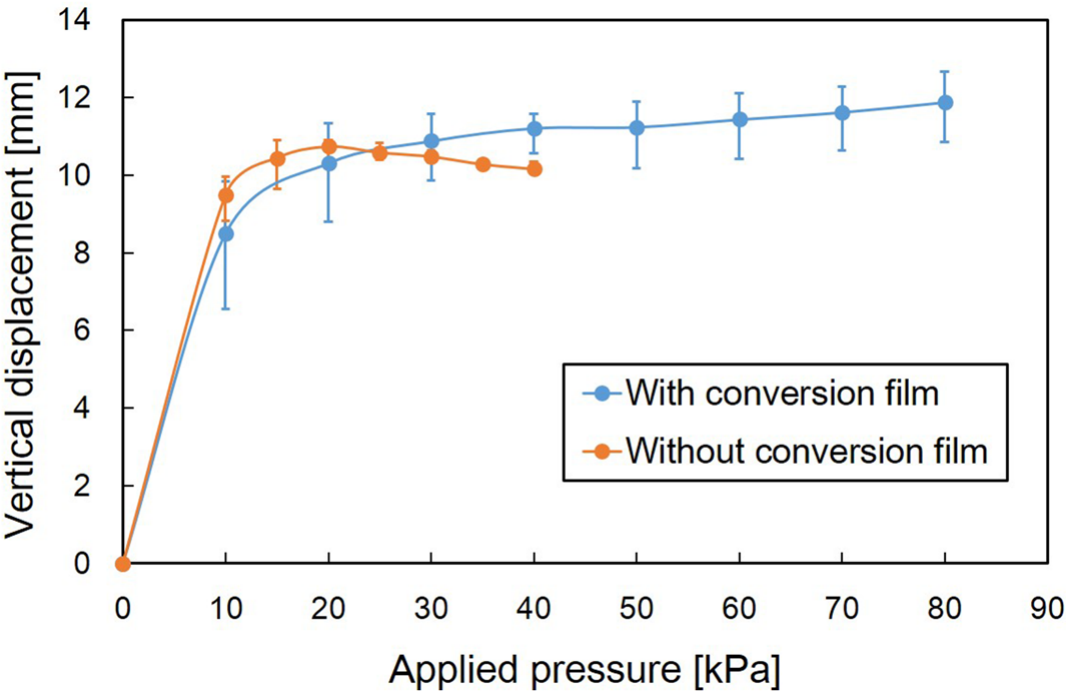
Relationship between applied pressure and vertical displacement of different bending-type PBAs without a load. The vertical displacement without a load was measured 2 mm from a bonding part. The absolute error is used. The microactuator with/without conversion film could perform up to 80 kPa/40 kPa of driving pressure. Both microactuators achieved more than 10 mm of the vertical displacement.

The displacements of two different bending microactuators, with and without the conversion mechanism, are evaluated in Fig. 4. Both microactuators were fabricated with Si ribs under the base film (Fig. 3a, b) and were characterized with respect to vertical displacement. The tip of the developed microactuator was 10 mm from a bonding part of the films. A structure extending from a bonding part to a tip is regarded as a force transmission structure. A 2 mm long structure from a bonding part is defined as the structure of the microactuator itself in this study. The vertical displacement without a load was measured 2 mm from a bonding part in Fig. 4. The microactuator with/without conversion film could perform up to 80 kPa/40 kPa of driving pressure, respectively. Some pneumatic balloons of the microactuators inflated too much and broke over 80 kPa/40 kPa. Relatively large dispersion was observed for the microactuator with conversion film around 10 kPa. It is likely that the dispersion was caused by the slight difference in timing at the time of contacting between the balloon and the film. The output is sensitive to the timing of the contacting. The error bar increased in the high-pressure range because of the influence of dispersion on the characteristics of the balloon membrane. The bending microactuators with/without the conversion mechanism showed a similar performance; their vertical displacement increased steeply when the driving pressure was increased to 10 kPa. The vertical displacement of the microactuator without the conversion film reached the highest point at 20 kPa and then decreased gradually while the driving pressure was increased up to 40 kPa. On the other hand, the increase in displacement exhibited by the microactuator with the conversion film stopped and converged to a certain displacement as the driving pressure was increased from 10 kPa. A detailed analysis shows that the vertical displacement of the microactuator with the conversion film stopped increasing at a lower driving pressure. The largest displacement observed for the microactuator with the conversion film was a little bit smaller than that generated by the microactuator without the conversion film. The deformed conversion film generated an elastic restoring force. No inflating balloon reached the conversion film at low applied pressures in Fig. 4. It follows from this analysis that the inclusion of a conversion film restricts the bending motion when an inflating balloon does not push on the conversion film.

**Figure 5:**
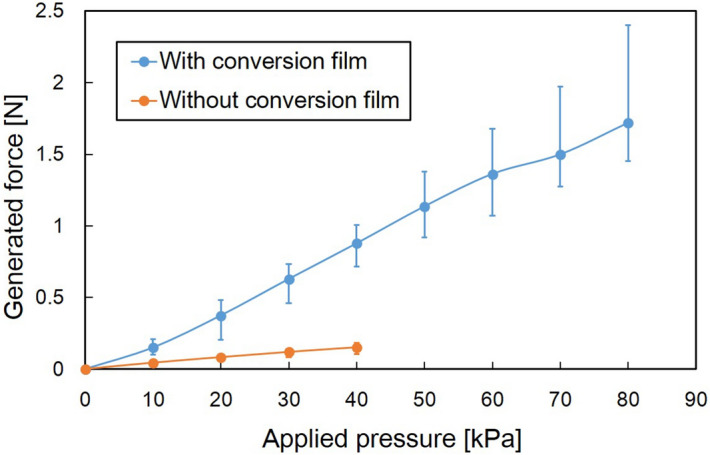
Relationship between applied pressure and generated force of different bending-type PBAs. The generated forces of the bending microactuators with/without the conversion mechanism were evaluated. The generated force was measured at a position 2 mm from a bonding part when the vertical displacement was 2 mm. The generated force increased in accordance with the applied pressure. The microactuator with a conversion film achieved a maximum force of 1.72 N at 80 kPa, whereas the microactuator without a conversion film achieved a maximum force of 0.15 N at 40 kPa.

Next, to evaluate the vertical displacement, the generated forces of the bending microactuators with/without the conversion mechanism were evaluated, as shown in Fig. 5. Fig. 5 shows the generated force measured at a position 2 mm from a bonding part which is defined as the microactuator structure itself in this study. The force was measured when the vertical displacement was 2 mm in Fig. 5. The generated force increased in accordance with the applied pressure. The microactuator with a conversion film achieved a maximum force of 1.72 N at 80 kPa of driving pressure. In contrast, the microactuator without a conversion film achieved a maximum force of 0.15 N at 40 kPa of driving pressure. The force generated by the microactuator with a conversion film was approximately six times as large as the force generated by the microactuator without a conversion film when 40 kPa was applied. In addition, the response time of the microactuator was evaluated. The rise time was 0.4 seconds when 80 kPa was applied. The microactuator system including peripheral fluidic supply took less than 0.4 seconds because the response time of electropneumatic regulator was estimated less than 0.1 seconds.

## Discussion

The conversion mechanism was designed for the high-output bending motion of a soft inflatable microactuator. The maximum driving pressure could be increased to 80 kPa, hence, the largest generated force was increased to 1.72 N. The generated force with the conversion mechanism could be approximately six times as large as that for the microactuator without the conversion film at the same driving pressure (40 kPa). The force density of the presented microactuator (16 mm × 30 mm × 850 μm) reached 4.2 mN / mm^3^ as a result of the improvement. The generated force was measured when the vertical displacement was 2 mm in Fig. 5. The vertical displacement during the force evaluation was set in the consideration of medical application for lifting an organ. This study also measured the generated force when the vertical displacement was 10 mm. The microactuator with the conversion film generated the largest force of 0.4 N at the tip at 80 kPa when the vertical displacement was 10 mm. These loads applied in the force measurement are equivalent to one and a half hundred grams/tens grams by simple weight conversion, respectively.

**Figure 6:**
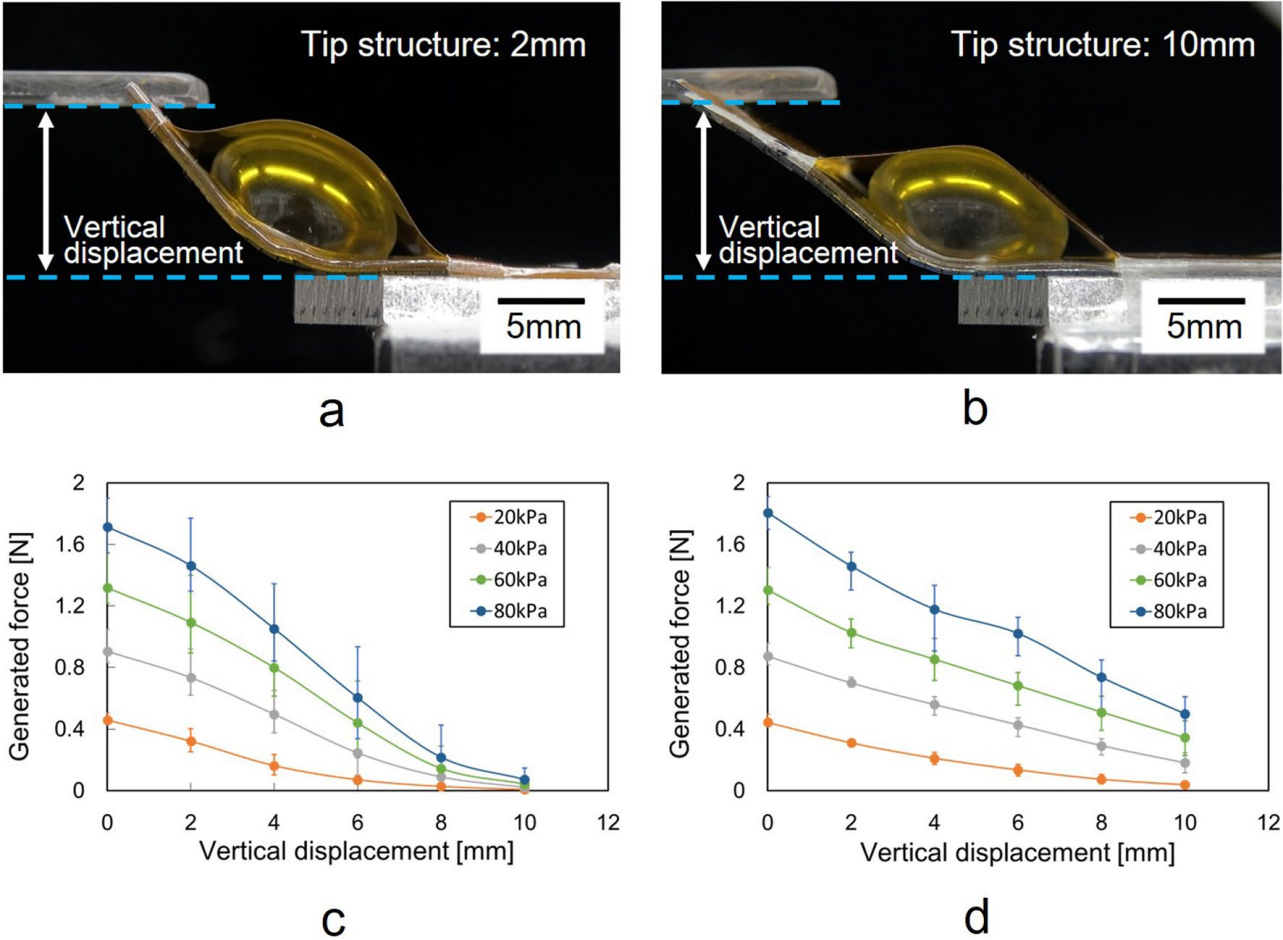
Performance characteristics of the bending microactuators with the conversion mechanism. (**a**) Side view of the bending microactuator whose tip is 2 mm from a bonding part of the films. (**b**) Side view of the bending microactuator with 10 mm long tip. (**c**) Performance characteristics of the microactuator of (**a**). (**d**) Performance characteristics of the microactuator of (**b**). Performance characteristics shows relationship between vertical displacement and generated force depending on applied pressures by combining results in Figs. 4 and 5. The generated force increased as the vertical displacement decreased. Both generated force and vertical displacement increased at higher applied pressure. The generated force curve in (**c**) deviated downward from the linear relationship in the high pressure range. The reachable distance of the microactuator can be increased by extending the tip structure.

Furthermore, the performance characteristics of the actuator with the conversion mechanism are summarized in Fig. 6, which combines the results of Figs. 4 and 5. The tip of the microactuator was 10 mm from a bonding part of the films. The structure of the microactuator itself was again defined as the structure spanning the 2 mm from the bonding part. Photographs of the side views of the microactuators are shown in both Fig. 6a, b. Fig. 6c, d show the performance characteristics of the actuator at positions 2mm and 10mm from the bonding part. A tip of the microactuator in Fig. 6a was cut at 2mm. The generated force increased as the vertical displacement decreased in both Fig. 6c, d. Both generated force and vertical displacement increased at higher applied pressure. The evaluation results are summarized in Table 1. For instance, Fig. 6d shows that a maximum force was generated at 0 mm of vertical displacement and the force became minimum at a maximum vertical displacement. At the same driving pressure, the maximum generated forces were almost the same at the positions of 2 mm and 10 mm from the bonding part (see Fig. 6c, d). As a result, a base film with ribs was stiff enough to transmit force from a bonding part to the tip of the actuator, as designed. The generated force in Fig. 6d linearly decreases with increasing vertical displacement. In contrast, characteristics of generated force curve in Fig. 6c deviated downward from this linear relationship in the high pressure range. The reachable distance of the microactuator with a 2 mm long tip structure is less than that of the microactuator with a 10 mm long tip structure. The influence of the limit of the reachable distance is noticeable once the vertical displacement in Fig. 6c increases to 4 mm.

**Table 1:** Stiffness estimation of the microactuator according to several pressure levels.

Pressure *P*_*i*_ [kPa]	Maximum force *F*_*max*_ [mN]	Maximum displacement *h*_*max*_ [mm]	R^2^	Stiffness *k* [N/m]
20	444	10.03	0.958	44.3
40	874	12.35	0.996	70.7
60	1,304	13.46	0.991	96.9
80	1,808	13.83	0.991	130.7

Table 1 estimates the stiffness of the bending microactuator with the conversion mechanism. Approximately linear relationships between the stiffness and the driving pressure were obtained by fitting the characteristics of Fig. 6d. The coefficients of determination are also shown. The stiffness k of the structure in Table 1 was calculated from the slope of the approximate straight lines. The stiffness was calculated from the slope of the approximately straight lines. The stiffness was calculated as follows:

$$k\left| {_{{P_{i} }} = \frac{{F_{max} }}{{h_{max} }}} \right|_{{P_{i} }} \quad \left[ {{\text{N}}/{\text{m}}} \right]$$

*k*: Stiffness of the PBA, *F*: Generated force, *h*: Vertical displacement, *P*_*i*_: Applied pressure.

Consequently, the structure of the microactuator increases its stiffness according to the driving pressure as shown in Table 1. We may, therefore, reasonably conclude that the conversion mechanism could contribute to the high-output bending motion especially in comparison with the same structure without the conversion film. In fact, the microactuator with the conversion film could generate a maximum force of 1.72 N at 80 kPa, whereas the microactuator without the conversion film generated a maximum force of 0.15 N at 40 kPa. The force density of the presented microactuator (16 mm × 30 mm × 850 μm) reached 4.2 mN / mm^3^ as a result of the improvement. As prospects, in addition to the first fundamental characterization in this paper, further performance characterization will be expected to allow optimum design in response to the specific requirements.

## Methods

### Fabrication process of the actuator with the conversion mechanism

Pneumatic balloons of most of conventional PBAs were fabricated by two-dimensional molding of polydimethylsiloxane (PDMS, Silpot 184, Dow Corning Inc.). However, KE-1606 (Shin-Etsu Chemical Co., Ltd.) which is a kind of silicone rubber, as the main material was employed instead of PDMS in this study. KE-1606 is characterized by attractive physical properties of elongation. The superior performance of elongation improves the expansion of balloon to resist pressure. Thus, the proposed device made of KE-1606 was expected to generate a considerable force.

**Figure 7:**
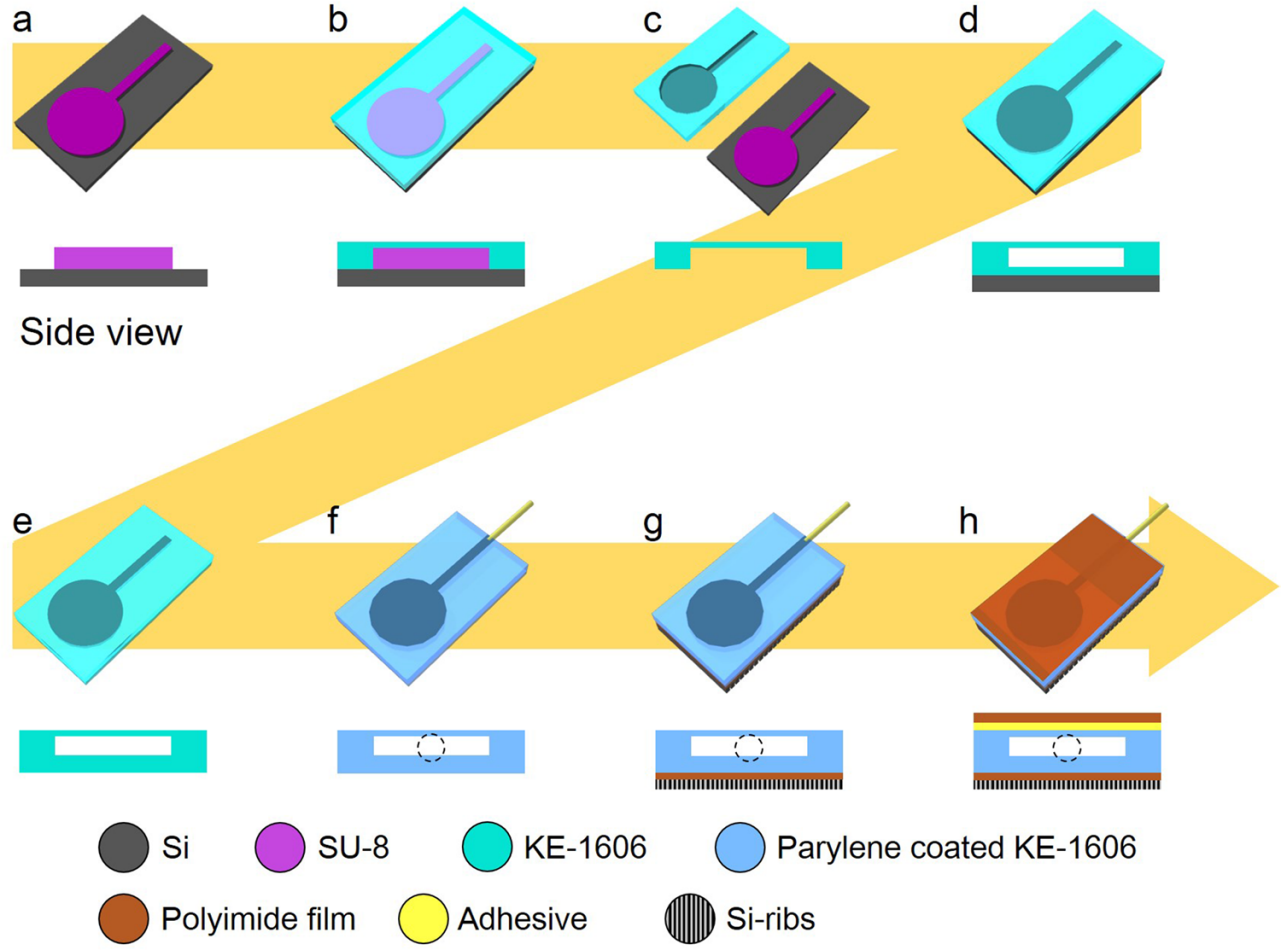
Fabrication process. (**a**) An SU-8 mold for PBA was fabricated on a Si wafer. Then, parylene (Parylene C, Specialty Coating Systems Inc.) was deposited on the substrate so that the KE-1606 could be easily removed. (**b**) The KE-1606 was spin-coated at 2000 rpm on SU-8 mold and thermally cured at 85 °C for 10 min. (**c**) After curing, the KE-1606 film with a transferred pattern was peeled off from the mold. (**d**) The patterned KE-1606 film was placed on another flat KE-1606 film and adhered by using surface activation (vacuum ultraviolet light). (**e**) The bonded KE-1606 film was peeled off from the wafer. (**f**) An interconnection tube was connected to create an air inlet. Then, the KE-1606 was coated with parylene C to reduce the frictional resistance when in contact with the polyimide film (Kapton Polyimide films, DuPont de Nemours, Inc.). Furthermore, this coating helps to improve the sealing performance. The KE-1606 film is a porous material and gas permeable. The parylene coating can seal the porous structure of the KE-1606 and allows efficient inflation of the balloons. (**g**) Si ribs were formed by dicing 400 μm thick Si wafer. The width of each rib was 950 μm and gap between adjacent ribs was 50 μm. The ribs were bonded to the 25 μm thick polyimide film and integrated into the device. (**h**) The 25 μm thick polyimide film, the force conversion film over the balloon structure of the PBA, were bonded at both ends on the base structure.

Figure 7 describes the fabrication process of the inflatable microactuator with the conversion film. An SU-8 mold for PBA was fabricated on a Si wafer (Fig. 7a). The mold thickness, which decided the height of the channel and cavity for the PBA, was 65 μm. Then, parylene (Parylene C, Specialty Coating Systems Inc.) was deposited on the substrate so that the KE-1606 could be easily removed. The KE-1606 was spin-coated (2000 rpm) on SU-8 mold and thermally cured at 85 °C for 10 min (Fig. 7b). After curing, the KE-1606 film with a transferred pattern was peeled off from the mold (Fig. 7c). In parallel, another KE-1606 film was prepared by spin-coating and curing on a bare Si wafer. The patterned KE-1606 film was placed on another flat KE-1606 film and adhered by using surface activation (vacuum ultraviolet light) (Fig. 7d). A part for a cavity of the balloon, which should not be bonded, was covered with a polyimide stencil mask so that the masked part was not exposed to the UV irradiation. The bonded KE-1606 films were peeled off from the wafer after bonding (Fig. 7e). Here, an interconnection tube was connected to create an air inlet (Fig. 7f). Then, the KE-1606 was coated with parylene C (0.1μm thick) to reduce the frictional resistance when in contact with the polyimide film (Kapton Polyimide films, DuPont de Nemours, Inc.). This parylene coating helps to improve the sealing performance as well. The KE-1606 film is a porous material and gas permeable. The parylene coating can seal the porous structure of the KE-1606 and allows efficient inflation of the balloons. On the other hand, Si ribs were formed by dicing 400 μm thick Si wafer. The width of each rib was 950 μm and gap between adjacent ribs was 50 μm. The ribs were bonded to a 25 μm thick polyimide film and integrated on the back side of the device (Fig. 7g). Finally, the 25 μm thick polyimide film, the force conversion film over the balloon structure of the PBA, were bonded at both ends on the base structure (Fig. 7h).

### Experimental setup

**Figure 8:**
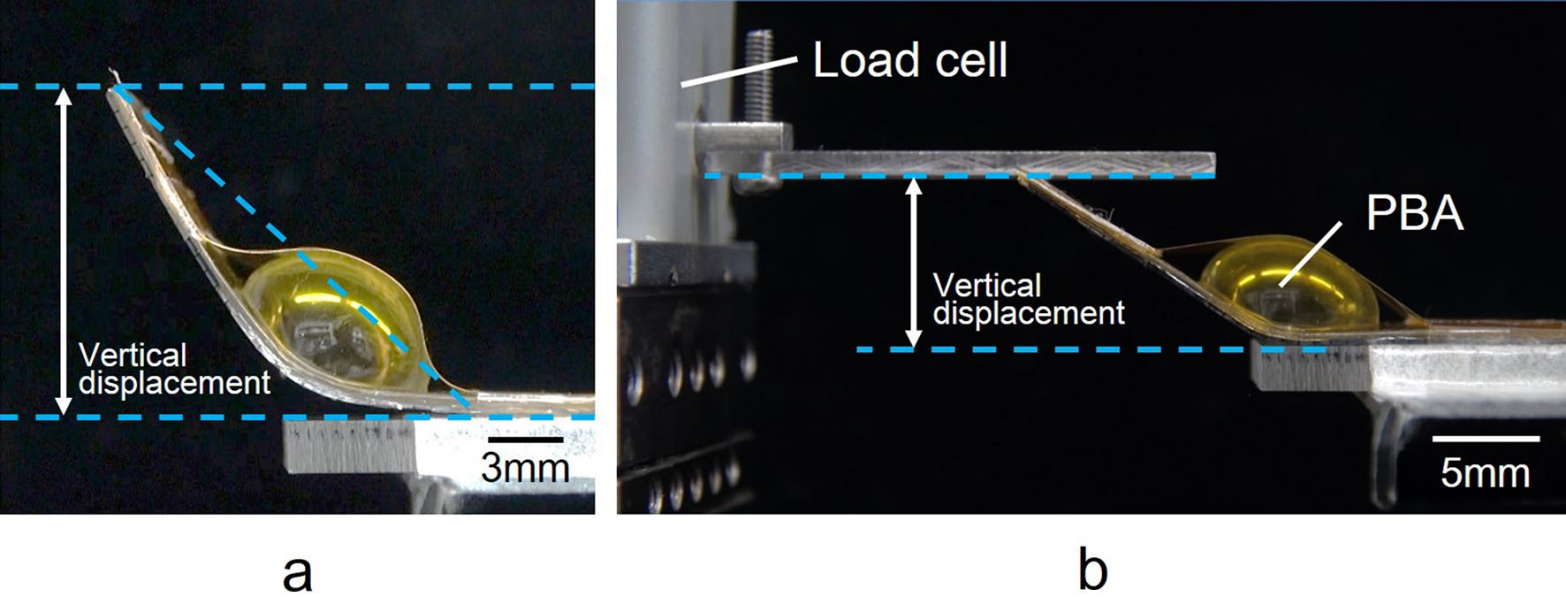
Experimental setup. (**a**) Measurement of the vertical displacement of the unloaded microactuator using a side view. (**b**) Measurement of the generated force. The vertical displacement in (**b**) was set to 10 mm. The generated force was measured by a load cell.

The vertical displacement and the generated force of the microactuator were evaluated in this study according to the applied pressure. The microactuator was driven by the applied air pressure controlled by a syringe pump (PUMP33, Harvard Apparatus). The pressure flow rate was set to a constant 20 ml/min. The bending motion of the unloaded microactuator was observed from the side direction to measure the vertical displacement. The displacement measurement was determined from the vertical height from the initial position line of the microactuator to the tip of the microactuator, as shown in Fig. 8a. Figure 8b shows the experimental setup for the measurement of the generated force of the microactuator according to the applied pressure. The vertical displacement in Fig. 8b was set to 10 mm. The generated force at the tip of the microactuator was measured using a load cell (LVS-500GA, Kyowa Electronic Instruments Co.) which combined with a strain amplifier (DPM-911B, Kyowa Electronic Instruments Co.). In addition, the generated force of the microactuator in each displacement was measured by moving the load cell to change the distance of the contact point with the microactuator.

## Supplementary information

Supplementary video

## Data Availability

All data generated or analyzed during this study are included in this published article.
